# Experiences of primary health care practitioners dealing with emergencies – ‘We are on our own’

**DOI:** 10.4102/phcfm.v15i1.3553

**Published:** 2023-01-31

**Authors:** Meghan Botes, Richard Cooke, Judith Bruce

**Affiliations:** 1 Department of Nursing Education, Faculty of Health Sciences, University of the Witwatersrand, Johannesburg, South Africa; 2 Department of Family Medicine and Primary Care, Faculty of Health Sciences, University of the Witwatersrand, Johannesburg, South Africa

**Keywords:** experiences, primary health care practitioners, emergency care, acute care, emergency management

## Abstract

**Background:**

Primary health care (PHC) focuses on health promotion and disease prevention; however, acute episodes and emergencies still occur at this level of care. The World Health Organization (WHO) proposes strengthening emergency care at a PHC level as a way of lessening the burden of disease on the overall health system. It is not known how health care practitioners at the PHC level experience management of emergencies.

**Aim:**

To explore and describe the experiences of PHC practitioners dealing with emergencies at PHC facilities in Gauteng, South Africa.

**Setting:**

The study was conducted in the District Health Services of Gauteng province in South Africa, including clinics, community health care centres and district hospitals.

**Methods:**

Using a qualitative approach, semi-structured interviews were conducted with a purposively selected sample of professional nurses and doctors from various levels of the district health care system. Data were transcribed and analysed using qualitative thematic analysis.

**Results:**

Various themes were identified related to the individual confidence and competence of the PHC practitioner, the team approach, the process of role and task allocation and the need for training.

**Conclusion:**

The study provided a voice for the needs of health care practitioners dealing with emergencies at the PHC level. The designing of a targeted and contextually appropriate approach to emergency care training of health care practitioners in the PHC setting that improves team dynamics and team performance, is recommended.

**Contribution:**

The insights of PHC practitioners dealing with emergencies contribute contextual relevance to any strategic improvement of care at this level.

## Introduction

Described as a situation involving a serious and unexpected, life-threatening illness or injury, medical emergencies are time sensitive in nature and require immediate action.^1^ A goal of emergency care is to deliver immediate care to patients with life-threatening illness or injury^1^ and where practical, at the first contact or first line of care. The first line of health care is primary health care (PHC), which provides an essential foundation for managing health emergencies and their associated risks.^2^ However, the ability to manage emergencies at this level of the health system is under scrutiny, leading to claims that emergency care in PHC settings has been neglected and is unable to provide communities access to good quality care.^2,3^ Specialised emergency care is often found only at higher-level institutions, and as a result, these hospital emergency departments are flooded with a high patient load, and a significant burden is put on hospital resources.^4^

The adoption of the World Health Assembly Resolution regarding Health Systems and Emergency Care Systems has given credence to the call to improve emergency care access and availability, globally. This resolution underscores the important role of strengthened emergency care systems in reducing the burden of disease from acute illness, trauma and emergencies in populations with varied socioeconomic circumstances, particularly in middle- and lower-income countries.^5^ The clarion call to the World Health Organization (WHO) and governments all over the world is to prioritise accessibility and provisioning of emergency care at all levels. In the same vein, and with greater specificity, the 72nd World Health Assembly^5^ deems the prioritisation of emergency services at a PHC level as a highly cost-effective way to ease the overall burden of disease on the health care system.

The vital components of effective emergency care at the PHC level include early recognition, using a team approach, clinical decision-making and intervention to stabilise patients and prevent further deterioration or complications. Inherent in this statement is the expectation that PHC practitioners possess the knowledge and skills to execute these tasks. However, PHC practitioners reportedly lack specialised training to deal with medical emergencies.^6,7^

The public health care system in South Africa is divided into four levels of care, each level offering a different package of services. Level one facilities include PHC clinics, community health care centres and district hospitals. At level two, regional hospitals provide basic definitive care with referral to tertiary and academic hospitals with specialist services in level three. Level four hospitals include all central and specialist hospitals.^8^

It is important to note the difference in skills mix in the team at the different levels of care. In sub-Saharan Africa, PHC services are considered nurse-led with support from care workers, doctors and allied medical professionals.^9^ It is in this context that we must frame our evaluation of emergency care at this level to fully appreciate the nuances of the team providing this service. Leading and decision-making in higher-level facilities are presumed to be the role of a doctor and in rare circumstances, a specialist nurse.^10^ Nurses in PHC may not be prepared to lead as the first responders to emergency situations. However, their leadership is essential at the PHC level, where a doctor, specialist nurse or specialised team is not always available to take the lead. It is therefore vital to ensure that all health care professionals, including nurses, clinical associates and doctors, practising in PHC facilities, are equipped and confident to lead a team in the emergency care process. The WHO guidelines for essential trauma care highlight the need for continuous education and training in emergency skills.^11^

Health care practitioners are thus expected to be able to manage emergencies at all levels of the health care system. With the roll-out of improvement strategies such as the Ideal Clinic Framework and the need for improved access as a goal of the proposed National Health Insurance scheme, understanding the current context of emergency care is vital.^12^ The emic perspective of PHC practitioners and their experiences of dealing with emergencies in the context of PHC are unexplored and therefore not known. The study aimed to illuminate multiple perspectives of emergency care in PHC settings with a focus on exploring the experiences of a variety of PHC practitioners and facility managers dealing with emergencies. The objective of the study was to explore and describe the experiences of health care practitioners in dealing with emergencies at PHC facilities in the Gauteng province, South Africa.

## Methods

### Study design

An exploratory descriptive qualitative method was adopted to gain insight into the experiences of PHC practitioners, managing patients in need of emergency treatment.

### Context

The study was conducted in one province in South Africa, focussing on the three levels of the district health services, namely clinics, community health care centres and district hospitals. The province of interest, Gauteng, is divided into five districts with a total of 392 PHC facilities. Primary health care in this context is largely nurse-led with support from medical doctors and clinical associates increasing at higher level facilities and allied health services provided at larger community health care clinics and district hospitals. The district health services focus on primary care and prevention of disease and function in a variety of settings from urban to semi-urban and semi-rural communities within the Gauteng province. While emergency care is not the focus in the context of primary health care, it still requires the mangement of nurses, doctors and clinical associates in resource constrained settings with overwhelming patient numbers and long delays in emergency transfers.

### Selection of participants

The population included all health care practitioners dealing with emergencies at a PHC level at the time of data collection. Purposive sampling was used to select a sample of health professionals working in a variety of PHC facility types and districts.

Inclusion criteria for participants included the following:

- A registered health care professional.
- Dealing with emergencies at a PHC facility.

Facilities were selected from a list of all PHC facilities in Gauteng by using an automated random selection formula on a prepared excel spreadsheet. A clinic, community health care centre and district hospital were randomly selected, from which particpants were recruited. The process was repeated until data saturation was reached. The final sample included 22 doctors and professional nurses (*n* = 22) from nine randomly selected facilities across four districts.

### Data collection

For data collection purposes, semi-structured interviews were conducted; participants were asked to describe their experiences of managing emergencies at a PHC facility with the opening question ‘What is your experience in dealing with emergencies in a primary health care facility?’. An interview guide was used to elicit information about practical issues regarding emergency care, and probing sub-questions were used for elaboration.^13^ Interviews were audio recorded and transcribed. Interviews were conducted on site by the researcher at the selected facility and all interviews were conducted in English and lasted between 30 and 45 min each.

### Data analysis

Data were analysed using thematic analysis with the aim of identifying important aspects of the content, to present them clearly and effectively.^13^ A step-by-step process was used to organise the interview data, and uploaded into a qualitative data analysis software system (MAXQDA Analytics Pro2018). The steps of thematic analysis included:

- Familiarisation with the data by reading and re-reading of transcripts.
- Concepts and patterns were identified and coded.^13^
- Codes were then organised into categories that were built into overarching themes for interpretation.^14^

### Trustworthiness

Trustworthiness, described by Graneheim and Lundt (2003), is a concept employed in qualitative research to ensure that the findings of a study are presented in a way that is a true representation of the data collected. The findings were analysed and presented in an unbiased manner allowing for readers to formulate their own interpretation; this was done by including adequate representation of quotations from the data. The use of a qualitative data analysis software package, MAXQDA^®^, assisted in ensuring good data management and organisation. A clear audit trail was kept in an electronic format and the methods of analysis are transparent.^15^ Transparent analysis allows for reflexivity as the researcher continuously assessed own bias and assumptions to uphold the veracity of the findings. The selection of health care practitioners at the coalface of emergency care at the PHC level ensured that appropriate experiences and characteristics required to answer the research question were met, ensuring the credibility of the study. Dependability was achieved by considering and including in the discussion of findings, new insights, policies, changes or inconsistencies encountered during the data collection process. The supervisors of this work monitored the data collection, data management and analysis to ensure the accuracy of findings and their coherence with themes and sub-themes. A rich description of the context, culture and characteristics of the participants, methods of data collection and analysis are provided to account for transferability.^16^

### Ethical considerations

Ethical clearance and approval to conduct the research were granted by the Human Research Ethics Committee (Medical) of the University of the Witwatersrand (No. M171115). Written informed consent was obtained from health professionals for participating in the study and for the digital recording of the interviews. Confidentiality and anonymity were maintained for the participants and the facilities through allocating pseudonyms or numbers to the participants and transcripts. The information letter informed participants of their choice to participate and option to withdraw at any stage of the research process.

## Findings

### Demographic profile of participants

Of the participants, 63.63% (*n* = 14) were doctors and 36.36% (*n* = 8) were nurses. While the District Health Care system is largely staffed by nurses, clinical management positions in Community Health Care centres and district hospitals were held largely by doctors. This accounts for the disproportion in representation from the two professions; however, the findings showed largely, agreement between the perceptions and experiences of both professions and therefore the disproportion was not found to distort the findings.

Primary health care clinicians accounted for 54.54% (*n* = 12) of the sample and 45.45% (*n* = 10), facility managers. Table 1 summarises the demographic characteristics of the participants.

**TABLE 1:** Demographics of participants (*n* = 22).

Category	Frequency (*n*)	Percentage (%)
**Gender**		
Female	5	22.72
Male	17	77.27
**Race**		
Black	16	72.72
White	1	4.54
Indian	1	4.54
Coloured	2	9.09
Other	2	9.09
**Profession**		
Nurse	8	36.36
Doctor	14	63.63
**Field of expertise**		
Management	12	54.54
Non-management	10	45.45

The number of years working in the current facility and within the district health services ranged greatly and increased in participants who held management positions. This ensured that the experiences shared covered a vast range of professionals from the most junior to those who have had many years of experience.

Of concern is the absence of updated emergency skills training among these professionals; 50% (*n* = 11) have had no emergency training since their basic qualification, while 13.6% (*n* = 3) last received emergency skills training over 5 years ago. Only 36.6% (*n* = 8) of the participants had training less than 5 years ago as depicted in Table 2.

**TABLE 2:** Years of experience and last emergency training of participants (*n* = 22).

Category	Frequency (*n*)	Percentage (%)
**Years since last training in emergency management**		
5 or less	8	36.36
6–10 years	1	0.05
11 or more years	2	0.09
No training	11	50.00
**Years working in facility**		
5 or less	11	50.00
6 to 10	4	18.18
11 to 20	3	13.63
21 or more	1	4.54
Undefined	3	13.36
**Years working in district health services**		
5 or less	6	27.27
6 to 10	4	18.18
11 to 20	4	18.18
21 to 30	4	18.18
31 or more	1	4.54
Undefined	3	13.36

### Participant experiences

Three broad themes and eight subthemes (Table 3) related to the personal experiences of PHC practitioners dealing with emergencies emerged from the data. A common expressed need was for continuous training in emergency management; team performance and collaboration between practitioners were considered vital for optimal emergency management.

**TABLE 3:** Summary of themes and subthemes.

Themes	Subthemes
Personal experience in managing emergencies	Presentation of emergencies
	Perception of competence
	Independence versus isolation
Emergency management team	Team performance
	Team roles
	The pecking order
The call for training	Regular and rigorous training
	Nursing capabilities

#### Theme 1: Personal experience in managing emergencies

Participants were asked to describe their experiences in managing emergencies at a PHC facility. Giving their personal accounts, participants expressed feelings of confidence, or a lack thereof, and also shared the types, severity and frequency of emergencies they encounter daily. Variation in the nature of emergencies would, understandably, test practitioners’ feelings of confidence and competence as cases present at their facilities.

**Presentation of emergencies:** At a PHC level, practitioners are likely to encounter a broad range of emergency conditions. The types and frequency of emergencies frame the experiences of the health care professional. Common conditions include traumatic injuries, medical emergencies, chronic illnesses, hypertension and diabetes mellitus – often uncontrolled. Patients may present with deteriorating or complicated chronic illness or sudden traumatic life-threatening events requiring efficient emergency care as expressed (or observed) by participants:

‘Things like trauma, elevated, uncontrolled hypertension or uncontrolled diabetes that kind of thing.’ (Participant 22, Male Doctor Community Health Care centre)

‘Usually it will be a very sick patient, maybe [*he*] was very ill at home for a long time and then come here very sick.’ (Participant 19, Female, Registered Nurse, Primary Health Care clinic)

The frequency of emergencies differed according to the facility the participants worked at but the occurrence of emergencies whether frequent or rare remained a reality for all participants, highlighting the need for all health care professionals to be equipped and ready for the event of an emergency admission. Differences in the frequency of emergency cases were expressed as follows:

‘Firstly, we don’t get that many. It’s not a case of we have one every day.’ (Participant 18, Female, Doctor, Primary Health Care clinic)

‘I’ll say yes, because there’s never a day where we don’t have an emergency.’ (Participant 7, Female, Registered Nurse, Primary Health Care clinic)

**Perception of competence:** Study participants acknowledged a range of feelings from being confident and appreciating the learning opportunity that emergencies present to being discouraged and often unheard in circumstances of having to deal with emergencies on their own. Their own competence aside, participants also expressed their frustration with not having the capacity in terms of resources and much-needed support to deal with emergencies optimally, and the effect it has on them personally and on the patients. The following excerpts illustrate their lack of confidence in managing emergencies and the stress associated with it:

‘I don’t feel confident and then even the management, I think we need some training, in-service training to bring the confidence back, because we are not confident, because now we are compromising some of the things, so we don’t feel confident.’ (Participant 20, Female, Registered Nurse, Community Health Care Centre)

‘I sometimes feel like we are not well capacitated’ … ‘As long as you are in a health facility, as a healthcare provider, you must be capacitated … you must know. When anybody who walks in, when there is an emergency, you can’t stand there and say, call so and so, you have to be hands-on and my observation was, second thing, you know even, I’m just going to be honest and open with you, even some of the doctors, they don’t know what’s going on when it comes to emergency.’ (Participant 2, Female, Registered Nurse, Primary Health Care clinic)

‘You become so stressed in such a way that at times you think that you know what, I don’t want even to wake up to come to this facility because I’m going to be mad, you see, and then, even the staff, then they become very frustrated too.’ (Participant 10, Female, Registered Nurse, Community Health Care Centre)

**Independence versus isolation:** All level one, facilities fall under the umbrella of PHC or District Health Services. Registered nurses are often the highest level of health care practitioners available in these facilities and therefore need to function autonomously. For practitioners, being on their own in PHC facilities is not synonymous to being independent. While forced independence creates the opportunity for nurses to be autonomous and to acquire more knowledge, emergency care is underpinned by a team approach, and therefore working in isolation can be detrimental for patients in need of emergency care. Nurse participants added the following:

‘I’m basically here just as a nurse. You are at the top of the chain. So, you need to function independently in a way.’ (Participant 14, Male, Registered Nurse, Primary Health Care clinic)

‘I can say my experience is that we gain a lot of knowledge because we are on our own, the doctors are not here. So, we have to be more knowledgeable and then we experience different cases. So, we end up knowing some of the emergencies we aren’t being trained on how to manage them.’ (Participant 17, Female, Registered Nurse, Primary Health Care clinic)

#### Theme 2: The emergency management team

Participants are familiar with the deployment of a team in response to emergencies, notwithstanding the factors that may influence team functionality. A team approach to emergency care has proven its efficacy over decades of research and having a functional emergency team is vital for effective emergency care.^17^ Clearly defined roles, competent team members and functional team dynamics are all factors, which contribute to team performance. Team performance in relation to team roles and hierarchies within the emergency team emerged as subthemes.

**Team performance:** Acknowledging the interdependence between team members, participants shared contrasting experiences of PHC team performance and functioning. Team performance was generally viewed in a positive light despite concerns about confidence, competence and resources. Participants expressed their reliance on team members and its importance in dealing with emergencies, including their satisfaction with how the team worked together in the event of an emergency:

‘We are so supportive, we support each other like this, all of us, we work well together.’ (Participant 3, Female, Registered Nurse, District Hospital Emergency Department)

‘You know that always when there’s somebody else you can share ideas.’ (Participant 8, Female, Doctor, Primary Health Care clinic)

On the other hand, some cited the lack of teamwork and the expectation that emergencies are dealt with by doctors on their own, raising concerns about the level of competency of members of the team:

‘There’s no team. At least if they can do that it (training) will give them the idea that this is supposed to be done as a team and not just a doctor alone.’ (Participant 22, Male, Doctor, Community Health Care Centre)

‘It’s easier to manage emergencies when you’re the [*inaudible*] and when you have a team that is competent whenever an emergency comes everybody knows where they are running and what they are doing but when you have an emergency, and the level of incompetency is just; I don’t know.’ (Participant 15, Female, Doctor, Primary Health Care clinic)

‘[*T*]hey are not yet competent or confident enough to can manage that.’ (Participant 2, Female, Registered Nurse, Primary Health Care clinic)

Participants shared their experiences of the support available to the team during emergencies. Some participants described the availability of support from outside structures such as consultants and doctors from higher level facilities, while others struggled to receive support from colleagues within the facility as well as from other facilities:

‘Ja, there’s good support as long as you call, they will always give you advice especially if you say you are stuck ….’ (Participant 8, Female, Doctor, Primary Health Care clinic)

‘There is actually no one to consult with. You can call a doctor in Mamelodi and say look this is my problem what can I do but you try to avoid that because it takes a lot of time to get somebody on call or the registrar or somebody to say look, I need to talk to you about this. If it’s an emergency, it’s very difficult to get these people. There’s no one that’s sitting at the other end of the telephone just waiting for you to call. So, you try to avoid that and just do your best under the circumstances.’ (Participant 18, Female, Doctor, Primary Health Care clinic)

A decrease in support at night was also a concern raised by participants:

‘But sometimes it is in the middle of the night, and no one answers their phone then you just manage the patient as far as you can and try to transfer them and during the day we actually have more support …’ (Participant 5, Female, Doctor, District Hospital Emergency Department)

**Team roles:** Participants’ experience of team roles was conflated with that of task allocation, narrating how members of the team would be assigned tasks either at the beginning of the day or as the emergency care was being rendered. Being assigned particular tasks would seemingly define the role of the team member during emergencies, which differs from a hospital emergency department:

‘Unfortunately, it’s not like in the hospital. They actually have nurse 1, nurse 2, like in the hospital in the emergency room. Here, like here in our department, we are only three registered nurses for all these departments. When the emergency come, it means we have to divide ourselves and go in there, that this one is going to help the doctor with the resuscitate, this one is going to give us the drugs when we call, this one is going to phone for an ambulance or whatever.’ (Participant 20, Female, Registered Nurse, Community Health Care Centre)

‘At times I do timekeeping, at times I give medication, whatever the doctor wants, and then I give to the patient. You see, it depends on that day what role you are given.’ (Participant 3, - Female, Registered Nurse, District Hospital Emergency Department)

The nurse’s role in the emergency care team is debated as nurses are often assigned to the tasks that ‘aid’ the team leader. The role of the nurse in the team varied according to the level of care. At the more basic primary care facilities, nurses are expected to take the lead. When a multidisciplinary team is available, the lead role shifts to the doctor. In this regard, nurse participants had the following to say:

‘Yes, I take the lead because we, as I’ve said before, we have a delegation for the month. You’ll find that for that month you are the leader for emergency cases, ja, so I’m always seeing that when I’m allocated for emergency patients.’ (Participant 17, Male, Registered Nurse, Primary Health Care clinic)

‘When an emergency come, we call the doctor and then the doctor will come to emergency room and see what he can do for the patient.’ (Participant 20, Female, Registered Nurse, Community Health Care Centre)

**The pecking order:** There was a clear but contrasting hierarchy of status expressed by participants. The nurses were considered to be involved in assessment and interventions needed in emergency care in most of the facilities; however, this varied according to the level of care and the level of training of the nurse. At the district hospital level, nurses were involved in decision making and interventions during emergency care, while at primary care facilities nurses relied on the doctors more. Although doctors are not permanently available at many of the PHC clinics, it is often assumed that when the doctor is available, they should take on the leadership role. This hierarchy of status is firmly entrenched in the attitude of doctors:

‘The doctor usually takes the role of the team leader.’ (Participant 5, Female, Doctor, District Hospital Emergency Department)

‘You are at the top of the chain. So, you need to function independently in a way. You need to take decisions. So, I’d say the role basically is to lead the team.’ (Participant 14, Male, Registered Nurse, Primary Health Care clinic)

‘The registered nurses’ roles, they’re just to carry out orders, like to give medication or to put up drips, ja, like, but they can’t examine a patient or help.’ (Participant 10, Female, Registered Nurse, Community Health Care Centre)

#### Theme 3: The call for training

**Regular and rigorous training:** Central to the success of emergency care preparedness and delivery is a PHC workforce that is well trained, motivated, supported and appropriately resourced. A dire need for training was emphasised during the interviews with all participants. The frequency, scope and intensity of such training to achieve the desired emergency care skills resonated with all participants. Requirements specified by the Ideal Clinic Status policy, such as Basic Life Support (BLS), were considered essential for all health workers at PHC clinics. Primary health care practitioners interviewed described their need and desire for training in emergency care as follows:

‘No, it’s [*training is*] not adequate … So now they’ve taken here one, there one, there, so that they can …, and still it’s not enough because 85% in the clinic must be trained.’ (Participant 10, Female, Registered Nurse, Community Health Care Centre)

‘I think more regular training definitely for all staff members; for all staff members not only, doctors and sisters but also the assistants so that everybody has at their level regular training that everybody is up to scratch because you don’t know when an emergency is going to happen.’ (Participant 18, Female, Doctor, Primary Health Care clinic)

‘That would definitely help because they don’t even know that they’re supposed to be a team because when you do BLS they tell you that you have to work as a team. There’s a team leader and everybody has a role in the team, but it doesn’t happen.’ (Participant 22, Male, Doctor, Community Health Care Centre)

Participants expressed mixed views about the availability of resources for training; some facilities relied on the Department of Health or the clinic itself to provide training, while others were specifically innovative in sourcing funding for their own training. It was apparent from the sentiments expressed that many practitioners were not willing to take the initiative or pay for their own training as illustrated below:

‘So, and then the department does allocate money for training. Right now we’re opening an accredited BLS, you know, and then basically we are running it with our own money. We’re already training about 50 something, in the last 2 or 3 months, in BLS. You know we are trying to improve but the department doesn’t, doesn’t help after all. The budget they put for training every year disappear just like that, I mean, nobody go for training or for this and that, you know, it’s a mess-up like that.’ (Participant 22, Male, Doctor, Community Health Care Centre)

‘So, you rely on the clinic to provide training.’ (Participant 6, Female, Registered Nurse, Primary Health Care clinic)

‘Ag, we don’t have like specialised training. The trainings that I give the Sisters [*Registered nurses*] is just informal but then we take our self for training.’ (Participant 15, Female, Doctor, Primary Health Care clinic)

**Nursing capabilities:** Capability in nursing is generally understood as a combination of knowledge, skills and personal qualities for maximum effectiveness in not only routine and predictable situations but also in unpredictable situations such as an emergency. Aligned with the team performance sub-theme, some participants’ experience of nursing capability and competence in an emergency situation was not encouraging. Performing triage when a patient arrives and providing basic emergency care was particularly challenging. The multidisciplinary team expressed concerns about nursing competence and the nurses’ level of training:

‘The challenge the majority of the time is the equipment and even the abilities of the nurses, because usually there are three to four nurses working, but only one of them is able to do … to take a line, to do sutures.’ (Participant 21, Female, Doctor, Community Health Care Centre)

‘My views on emergency management at a primary level is I don’t think nurses are that much equipped to deal with certain emergencies, you know.’ (Participant 16, Female, Registered Nurse, Primary Health Care clinic)

Triage, mainly a health practitioner role, is an important process for the effective management of emergencies. The triage process for most of the facilities starts at the administration desk where the clerk or help desk assistant would be expected to triage patients for emergencies. Flaws in the triaging process were expressed as follows:

‘[*W*]e’ve got somebody on the information desk, she just screams “Sister Mpho, I’ve got a patient bleeding”, then they take the patient there, rush her straight into the emergency. So that’s the clerks and everybody will do the register and they’ll follow up the patient in there.’ (Participant 7, Female, Registered Nurse, Primary Health Care clinic)

‘There’s no proper, ja, so where I worked before the nurse would triage the patient as they walk in. So, here they walk in and they either go sit in male or female where the sister takes the vitals but then they write the vitals down and just put it in. The file is already at the bottom.’ (Participant 5, Female, Doctor, District Hospital Emergency Department)

The experiences of PHC practitioners of emergency care at this level are contextually nuanced by the types and frequencies of emergencies, and the efficiency of care relies on a number of factors including the need for a functional team approach and regular training.

## Discussion

The main findings of this study have broadened our understanding of how PHC practitioners experience the management of emergencies that present at different levels of the District Health System. The main presenting illnesses encountered and described by the participants included traumatic injuries, medical emergencies of various acuity levels, complicated chronic illnesses (diabetes and hypertension) and maternity emergencies. Similar to a local study by Obermeyer, the profile of patients includes young people free of chronic disease, presenting with acute illness or injury.^18^ Most of the conditions treated at the PHC level can be classified as lifestyle or chronic conditions; however, death and disability occur when these conditions deteriorate into acute illness leading to an emergency situation. Early recognition and treatment of these medical emergencies may assist in reducing the mortality and morbidity related to these common conditions.^3^

One of the key findings of this study is that the personal experiences of PHC practitioners are shaped by perceptions of their own competence or ability to deal with presenting emergencies and feelings of relative independence transposed with feelings of isolation. For particularly nurses in PHC settings, the lack of support enhances their feelings of isolation and being ‘on their own’, thus displacing their sense of autonomy. The Scope of practice (R2598 under the *Act of 1978* as amended) charges the professional nurse in South Africa with the responsibility of ‘diagnosing a health need’ and ‘executing a nursing care regimen’ – this applies to any health need, whether urgent or chronic. The nurse’s confidence and ability to lead an emergency care team are vital at this level of care where doctors are not always available. In this regard, the nurses in this study expressed their need for support and regular emergency care training in order to enhance their confidence and competence in dealing with emergencies.

Emergency care becomes increasingly advanced at level two, three and four institutions where different specialty services can provide definitive care for patients with acute injuries or illness as opposed to the level one PHC clinics, community health care centres and district hospitals where emergency care would be managed by professional nurses or junior doctors with no specialised training in emergency care.^8^ Given this difference, it would be important to capacitate nurses who are often at the forefront of emergency care in this context.

From a legislative and regulatory perspective, professional nurses are responsible for providing emergency care.^19^ Globally, this responsibility is often met with negative attitudes from other health care practitioners, uncertainty and a lack of confidence in the nurses’ skill to lead the team. These negative attitudes can lead to hesitancy and failure to act in an emergency situation^10^ and may be a result of the nurse’s role in the emergency care team being assigned as supplementary to the team leader in higher-level facilities.

Another key finding is the need for an effective team, where clear roles and embedded emergency care competencies are central to optimal team performance. A team approach to emergency care has proven its efficacy over decades of research.^17,20,21^ It is vital to ensure that health care practitioners are equipped and confident to lead a team in the emergency care process. In their development of a tool to measure the performance of emergency teams, Van der Haar et al. described the attitudinal aspects of team performance as most important, which include team satisfaction, commitment and trust in management.^22^ For optimal team performance, there must be trust and commitment from all members. This requires clear communication and assignment of roles to ensure adherence to emergency interventions and thus improving team performance.^17^ In this study, it was found that task allocation was used interchangeably with role allocation. Although task allocation is important in the emergency care process, role clarification must take place to establish leadership and clear communication.

Thematically conceived as the pecking order in the team, the nurse’s role in the resuscitation team is a contentious issue as nurses are often assigned to the tasks that ‘aid’ the team leader, which is mostly the doctor. Leading and decision-making are presumed to be the role of a doctor and in rare circumstances, a specialist nurse.^10^ However, for any health care practitioner to render effective emergency care, there must be a system of support that enables effective practice regardless of the context. Instead of supporting and developing the nurse’s leadership role, the doctors assume leadership even though they are not permanently available at the clinics. Role clarification and team leadership development are vital for emergency care in PHC contexts.

Current recommendations are that emergency training should be updated at least every 2 years to reduce the loss of skill over time.^23^ The call for training was an abundant finding that cut across several subthemes. Primary health care practitioners are concerned about their own and their colleagues’ levels of competence and the skills needed for dealing with emergencies. Concerned about the level of training and the competence of the nurses in their team, doctors felt that in the absence of support and resources, they were forced to assume much of the responsibility of emergency care along the care continuum. In some instances, a vital clinical responsibility, such as triage is not performed by nurses, which may be a function of either nursing capabilities that are lacking or resource constraints at PHC facilities.

Triage is an important process for the effective management of emergencies. The first response is a defining event for the patient and the rest of the system.^24^ The factors affecting the effectiveness of triage include the skills and capacity of the practitioner performing triage.^25^ This assumes that triage is performed by a qualified health care practitioner. The use of administrative assistants for triaging is concerning. For the first response to emergencies to be effective, a qualified health professional trained in recognising deteriorating patients or acute disorders should be allocated to triaging and prioritisation of patients presenting to the facility.

Overall, these perspectives formulate the basis of a resounding need for ongoing training as well as specialised training in BLS. The WHO guidelines for essential trauma care highlight the need for continuous education and training in emergency skills.^26^ There is an emphasis in the literature on a lack of specialised training for PHC practitioners dealing with medical emergencies.^6^ Although facility management and provincial government are relied upon to provide training, there is an opportunity for authorities to consider models for insourced emergency training or a way of incentivising continuous professional development that focuses on emergency care.

## Limitations of the study

At the time of data collection, there were no clinical associates employed at the selected PHC facilities. This cadre of health professionals may provide valuable insight from their own experiences in managing emergencies at a PHC level, and further studies should include their perspectives.

## Recommendations

Given the overwhelming call for PHC practitioners to be equipped with skills and knowledge in emergency care, rigorous, regular and targeted training programmes are essential; however, it is recommended that such training adopt a nuanced approach that takes into consideration the context, the presenting emergencies and the scope of practice of the practitioner. In particular, nursing practitioners must be sufficiently capacitated from triage to transfer to strengthen the emergency care response in nurse-led primary care. Improvement in team dynamics, team performance and clarification of team member roles are recommended outcomes of training.

## Conclusion

Strengthening emergency care at a PHC level protects the health care system by preventing the overload of higher-level facilities and provides the patient with efficient care at the frontline of health care access. There is a need for formal training programmes to capacitate PHC practitioners with the knowledge and skills to manage emergencies and leading emergency care teams at a PHC level. While access to emergency care is a constitutional right, level one facilities have fallen behind in the realisation of this ideal. Clarification of health care practitioners’ roles, as well as regular training and appropriate support for practitioners working at this level are vital to ensure that the first response to emergencies is effective, and to ensure that they do not feel isolated in their efforts to uphold the first line of care.
